# Knowledge, attitude and readiness toward telehealth among nursing staff: a cross-sectional study

**DOI:** 10.1186/s12909-025-07921-1

**Published:** 2025-10-21

**Authors:** Reda Hassan Hussien, Gellan K. Ahmed, Salah Omar Abdullah

**Affiliations:** 1 Department of Nursing Administration, Faculty of Nursing, Assiut University, Assiut, Egypt; 2 Department of Neurology and Psychiatry, Faculty of Medicine, Assiut University, Assiut, Egypt; 3 Department of Psychiatric and Mental Health Nursing, Faculty of Medicine & Health Sciences, Hodeidah University, Hodeidah, Yemen

**Keywords:** Attitude, Knowledge, Readiness, Telehealth, Nursing staff

## Abstract

**Background:**

Telehealth has emerged as a promising solution for improving global healthcare delivery by leveraging telecommunications and information technology in remote nursing practice.

**Objectives:**

This study aimed to evaluate the knowledge, attitude, and readiness of nursing staff toward telehealth.

**Methods:**

A cross-sectional study design was employed, conducted across three hospitals affiliated with Assiut University: El-Rajehy, Al Orman, and the Neurology and Psychiatry Hospital. A convenience sample of 250 staff nurses participated in the study. Data were collected using four tools: a demographic and work-related characteristics questionnaire, the Health Professionals’ Knowledge toward Telemedicine Technology scale, the Health Professionals’ Attitude toward Telemedicine Technology scale, and the Telehealth Readiness Assessment Tool (TRA).

**Results:**

The study identified a significant positive correlation between nurses’ knowledge and their attitude and readiness for telehealth. Multiple regression analysis revealed that knowledge was a significant predictor of both attitude and readiness. Nurses with good knowledge demonstrated a higher proportion of positive attitudes (47.8%) compared to those with poor knowledge (2.7%). Notably, nurses with poor knowledge were more likely to report low readiness for telehealth (58.0%) compared to those with good knowledge (26.8%).

**Conclusions:**

These findings highlight the critical role of knowledge in shaping nurses’ attitudes and readiness for telehealth. Nurses with higher levels of knowledge were more likely to exhibit positive attitudes and higher readiness for telehealth implementation. Based on these results, it is essential to develop and implement comprehensive educational programs to enhance nursing staff’s knowledge and attitudes toward telehealth.

**Supplementary Information:**

The online version contains supplementary material available at 10.1186/s12909-025-07921-1.

## Background

The healthcare landscape has undergone profound transformation with the strategic integration of telehealth technologies, particularly within nursing practice domains. Telehealth, conceptualized as the systematic utilization of telecommunications technology to deliver healthcare services remotely, has emerged as a pivotal solution for addressing contemporary global healthcare challenges, including accessibility disparities and critical workforce shortages [1]. This technological paradigm enables healthcare providers to deliver comprehensive care through diverse platforms, encompassing video consultations, remote monitoring devices, and advanced telecommunication technologies, thereby fundamentally restructuring the traditional healthcare delivery framework [2]. For example, The COVID-19 pandemic served as a catalyst highlighting telehealth's critical importance for maintaining healthcare continuity during global health emergencies. Research documented significant increases in psychological problems among both healthcare workers and patients [3, 4] that could have been substantially reduced through effective telehealth implementation via remote psychological support services, mental health consultations, and continuous monitoring of healthcare workers' wellbeing.

The clinical significance of telehealth has been substantiated through extensive empirical research, particularly regarding its capacity to transcend geographical barriers and enhance healthcare accessibility. Contemporary evidence demonstrates that telehealth facilitates comprehensive diagnosis and management of chronic diseases while enabling real-time physiological monitoring, capturing critical health parameters that may be inadvertently overlooked during conventional clinical encounters [2]. Furthermore, telehealth readiness, operationally defined as the comprehensive preparedness of users, healthcare organizations, and healthcare systems to implement telehealth applications effectively, has been identified as a fundamental determinant of successful implementation [5].

The theoretical underpinning of telehealth is anchored in the Technology Acceptance Model (TAM), which provides a robust framework for understanding the factors that influence healthcare professionals’ adoption of telehealth technologies. TAM posits that perceived usefulness and perceived ease of use are fundamental determinants of technology acceptance and behavioral intention to use technology [6]. Additionally, the Diffusion of Innovation Theory (DOI) complements this framework by explaining how telehealth innovations spread through healthcare organizations and professional communities [7, 8]. These theoretical models collectively provide the conceptual foundation for examining knowledge, attitudes, and readiness as key variables influencing telehealth adoption among nursing professionals.

Notwithstanding its considerable potential, telehealth integration encounters substantial implementation barriers. Empirical investigations have revealed that nursing personnel frequently lack adequate competency-based training and comprehensive information regarding the utilization of telehealth in patient-centred care delivery [9]. This knowledge deficit potentially compromises care quality standards and underscores the imperative need for evidence-based, comprehensive training programs [10]. Additionally, research has identified multifaceted challenges encompassing technological infrastructure limitations, data security vulnerabilities, and digital divide disparities, which collectively impede effective telehealth service implementation [11].

Previous scholarly investigations have emphasized the critical importance of organizational readiness and robust support systems in facilitating successful telehealth implementation. Specifically, studies conducted across diverse healthcare settings have demonstrated that factors including IT infrastructure capabilities, human resource allocation, and perceived ease of use significantly influence telehealth adoption patterns [12]. Moreover, healthcare providers’ attitudes and perceptions toward telehealth have been found to demonstrate strong correlations with their willingness to adopt these innovative technologies [13].

In resource-constrained developing countries, telehealth implementation encounters additional contextual challenges. For instance, in Egypt, while telehealth represents a promising solution to address essential healthcare deficits in rural regions, persistent issues including limited telenursing experience, organizational resistance to change, and inadequate specialized equipment continue to present significant implementation barriers [14]. These challenges underscore the critical necessity for comprehensive assessment of healthcare providers’ readiness and targeted, evidence-based interventions to support successful implementation.

Despite the substantial body of research on telehealth implementation, a significant knowledge gap persists regarding nursing staff’s knowledge, attitudes, and readiness toward telehealth, particularly within specific healthcare contexts. This gap is critically important as nurses occupy a central role in healthcare delivery and are frequently positioned at the forefront of implementing innovative healthcare technologies [15]. Previous studies have consistently emphasized the need for more comprehensive regional, national, and global research addressing these fundamental aspects among nursing professionals [16].

Research examining the interrelationships among knowledge, attitude, and readiness regarding telehealth among nurses has emerged as a critical area of scholarly inquiry due to the accelerating integration of digital technologies in healthcare delivery and nurses’ pivotal role in telehealth implementation. 

Although numerous studies have investigated telehealth competencies and educational interventions [17], substantial gaps remain regarding how knowledge, attitude, and readiness collectively influence telehealth adoption among nursing professionals. For example, a study by Hussain et al. (2023) demonstrated that educational brochures significantly improved nurses' knowledge, perception, and attitude toward telenursing [18]. However, Hilfida et al. (2023) emphasized that despite such interventions, the synergy among these factors is not well understood in practice [19]. Some research posits knowledge as a fundamental precursor to readiness [20, 21], while alternative perspectives suggest that readiness itself can drive proactive learning behaviors [22]. This theoretical controversy highlights the need for a nuanced understanding of these complex relationships, as insufficient readiness or knowledge can significantly impede telehealth uptake, resulting in suboptimal care delivery. The consequential implications of this gap include delayed telehealth implementation and compromised quality of nursing care in digital environments [23].

For the purposes of this study, knowledge is conceptualized as the cognitive understanding and comprehension of telehealth technologies and practices, attitude as the affective disposition and evaluative orientation toward telehealth utilization, and readiness as the comprehensive preparedness and willingness to engage in telehealth activities [24]. The theoretical relationships among these constructs are firmly grounded in established technology acceptance theories, including the Technology Acceptance Model and the Unified Theory of Acceptance and Use of Technology, which theoretically posit that knowledge and attitude significantly influence readiness and behavioral intention [25]. Understanding these complex interrelationships is essential for informing evidence-based educational strategies and organizational policies that foster effective telehealth adoption by nursing professionals [26, 27]. For example, Chike-Harris et al. (2021) emphasized the importance of immersive telehealth education in preparing graduate nursing students for digital care delivery [27].

Consequently, this study aims to comprehensively assess the knowledge, attitudes, and readiness toward telehealth among nursing staff, thereby addressing this critical research gap. Understanding these fundamental factors is essential for developing effective, evidence-based strategies to enhance telehealth adoption and improve healthcare delivery through technology-enabled nursing care. The anticipated findings will contribute significantly to the expanding body of literature on telehealth implementation and provide valuable, actionable insights for healthcare organizations, policymakers, and educators in developing targeted, theory-driven interventions to support successful telehealth integration in contemporary nursing practice.

## Methods

### Sample size calculation

The total number of nurses in the three hospitals was 560. Using the software EPI/Info, version 3.3, with a 95% confidence interval (CI), the estimated sample size was calculated to be 228 nurses. To account for potential dropout and non-response rates, the sample size was increased to 250.

### Study tools

1. Demographic and work-related characteristics questionnaire: Developed by the researchers, this tool collected information on age, educational level, marital status, department, and years of work experience.
2. Health Professionals’ Knowledge and Attitude toward Telemedicine Technology Scales:

Two scales were developed to assess health professionals’ knowledge and attitudes toward telemedicine, both adapted from previous studies and existing literature [28].

The Knowledge Scale consists of 10 yes/no items that evaluate the respondents’ understanding of telehealth technology. Scores range from 0 to 10, with scores below 5 (50%) indicating poor knowledge and scores above 5 (50%) reflecting good knowledge [29].

The Attitude Scale includes 23 items assessing attitudes across five domains: perceived relative advantages, compatibility, complexity of telehealth deployment, trialability of telehealth applications, and observability. Responses are measured on a 5-point Likert scale. Mean scores below 2.5 (50%) suggest a poor attitude, scores between 2.6 (51%) and 3.0 (60%) indicate a moderate attitude, and scores above 3.0 (60%) reflect a good attitude [29].

3- Telehealth Readiness Assessment (TRA) tool, designed to evaluate healthcare providers’ preparedness for implementing telehealth services. This assessment tool identifies areas where telehealth services are ready for deployment, highlights areas requiring enhancement, and establishes improvement priorities. The TRA encompasses five essential domains critical for successful telehealth implementation: core readiness, financial factors, operational aspects, staff involvement, and patient preparedness. The assessment employs a 4-point Likert scale ranging from 0 (not applicable) to 3 (definitely), with intermediate values of 1 (no/unsure) and 2 (somewhat). Score interpretation follows the instrument’s guidelines: scores of 50% or below indicate low readiness, scores between 50% and 75% represent moderate readiness, and scores exceeding 75% demonstrate high readiness levels [30].

### Procedure

The study was conducted from December 2023 to March 2024. The questionnaire was distributed three days each week throughout three work shifts. A pilot study was performed with 30 nurses to assess the questionnaire’s clarity, feasibility, and applicability. These nurses were excluded from the final sample. No modification was required.

Validation and Reliability of Instruments.

The instruments were translated into Arabic using a systematic forward and backward translation process. The validity of all instruments was assessed by five professors from the Faculty of Nursing at Assiut University. The reliability of the tools was evaluated using the Cronbach’s alpha coefficient test, yielding values of 0.86, 0.84, and 0.89 for the knowledge, attitude, and readiness scales, respectively.

### Statistical analysis

Data entry and statistical analysis were conducted using SPSS version 26.0. Descriptive statistics for categorical variables were presented as frequencies and percentages, while continuous variables were presented as means and standard deviations. The Shapiro-Wilk test was used to assess the normality of continuous variables. For categorical data, the Chi-square (χ²) test was used for group comparisons, with Fisher’s exact test applied when expected cell frequencies were less than five. Independent samples t-tests were used to compare means between two groups for continuous variables. One-way analysis of variance (ANOVA) followed by post hoc tests was performed to examine differences across age groups and hospital types. Pearson correlation coefficients were calculated to assess the strength and direction of relationships between continuous variables. Multiple linear regression analysis was performed to identify predictors of nurses’ knowledge, attitudes, and readiness toward telehealth. Prior to regression analysis, model assumptions were verified including multicollinearity assessed using variance inflation factor (VIF) values with all values less than 10 and tolerance values greater than 0.1 indicating acceptable levels, normality confirmed through examination of residual plots and the Shapiro-Wilk test, linearity verified through scatterplots of residuals versus predicted values, and homoscedasticity confirmed through visual inspection of residual plots and statistical tests. All statistical tests were two-tailed, with a significance level set at *p* ≤ 0.05.

## Results

### Demographic characteristics

Table 1 presents the socio-demographic characteristics of the 250 nurses in this study. Participants were categorized into two groups based on their scores on knowledge about telehealth: Group 1: Poor knowledge (*n* = 112) and Group 2: Good knowledge (*n* = 138). Significant statistical differences were observed regarding age, qualifications, years of experience, and hospital affiliation. The good knowledge group had a lower mean age and a lower frequency of nurses over 40 years (16.7%) compared to the poor knowledge group. Nurses with a diploma in nursing and those working in the neurology and psychiatry hospital had a higher frequency in the poor knowledge group. Additionally, nurses with more than 20 years of experience were more prevalent in the poor knowledge group.

**Table 1:** The socio-demographic characteristics of the nurses (N=250)

Variables	All nurses (*N* = 250)	Group 1 (*N* = 112)	Group 2 (*N* = 138)	*P* value
Age (Mean ± SD)	34.64 ± 7.34	35.84 ± 8.19	33.67 ± 6.43	0.020*
< 30 years	64(25.6%)	29(25.9%)	35(25.4%)	0.005*
30–40	126(50.4%)	46(41.1%)	80(58.0%)	
> 40 years	60(24.0%)	37(33.0%)	23(16.7%)	
Sex N(%)				
Male	75(30.0%)	39(34.8%)	36(26.1%)	0.134
Female	175(70.0%)	73(65.2%)	102(73.9%)	
Marital Status N(%)				
Married	196(78.4%)	93(83.0%)	103.(74.6%)	0.109
Unmarried	54(21.6%)	19(17.0%)	35(25.4%)	
Level of Qualifications N(%)				
Bachelor of nursing	85(34.0%)	18(16.1%)	67(48.6%)	< 0.001*
Diploma	165(66.0%)	94(83.9%)	71(51.4%)	
Years’ Experience: N(%)				
< 10 years	115(46.0%)	40(35.7%)	75(54.3%)	< 0.001*
10–20	85(34.0%)	38(33.9%)	47(34.1%)	
> 20 years	50(20.0%)	34(30.4%)	16(11.6%)	
Type of Hospital N(%)				
Neurology and Psychiatry hospital	86(34.4%)	47(42.0%)	39(28.3%)	0.042*
Orman hospital	82(32.8%)	29(25.9%)	53(38.4%)	
Rajehy hospital	82(32.8%)	36(32.1%)	46(33.3%)	

*Group 1: Poor knowledge*

*Group 2: Good knowledge*

* p significant, t Independent samples t-tests, the Chi-square (χ²)test for categorized

### Attitude and readiness toward telehealth

Table 2 reveals significant differences among the studied groups regarding nurses’ attitudes and readiness toward telehealth, as well as their respective subscales. In terms of attitudes, the good knowledge group exhibited a higher frequency of good attitude levels (47.8%) compared to the poor knowledge group (2.7%). The good knowledge group also had the highest mean scores for the subscales and total scores of nurses’ attitude. Regarding readiness toward telehealth, the poor knowledge group had a higher frequency of low-level readiness (58.0%) compared to the good knowledge group (26.8%). The total score and subscales for readiness toward telehealth had the highest mean values in the good knowledge group.

**Table 2:** Nurses' attitude and readiness toward telehealth (N=250)

Variables	All nurses (*N* = 250)	Group 1 (*N* = 112)	Group 2 (*N* = 138)	*P* value
Levels of nurses’ attitude *N*(%)				
Poor attitude	102(40.8%)	91(81.3%)	11(8.0%)	< 0.001*
Moderate	79(31.6%)	18(16.1%)	61(44.2%)	
Good attitude	69(27.6%)	3(2.7%)	66(47.8%)	
Nurses’ Attitude (Mean ± SD)				
Relative advantage	18.98 ± 5.99	14.98 ± 5.10	22.23 ± 4.53	< 0.001*
Compatibility	10.76 ± 2.99	9.02 ± 2.20	12.17 ± 2.81	< 0.001*
Complexity	12.20 ± 3.29	10.55 ± 2.66	13.54 ± 3.16	< 0.001*
Trial ability	11.83 ± 3.16	10.29 ± 2.85	13.08 ± 2.84	< 0.001*
Observability	7.40 ± 2.76	6.16 ± 2.24	8.41 ± 2.73	< 0.001*
Total of nurses’ Attitude score (Mean ± SD)	61.17 ± 13.54	51.00 ± 8.74	69.43 ± 10.86	< 0.001*
Levels of readiness toward telehealth N(%)				
Low	104(41.6%)	65(58.0%)	39(28.3%)	< 0.001*
Moderate	107(42.8%)	45(40.2%)	62(44.9%)	
High	39(15.6%)	2(1.8%)	37(26.8%)	
Readiness toward Telehealth (Mean ± SD)				
Core readiness	15.45 ± 3.59	14.89 ± 2.98	15.90 ± 3.97	0.027*
Engagement readiness	25.77 ± 5.51	24.35 ± 3.71	26.92 ± 6.40	< 0.001*
Structural readiness	22.19 ± 4.45	20.36 ± 3.56	23.67 ± 4.55	< 0.001*
Total of Readiness toward Telehealth (Mean ± SD)	63.35 ± 11.73	59.60 ± 7.74	66.40 ± 13.44	< 0.001*

*Group 1: Poor knowledge*

*Group 2: Good knowledge*

* p significant, t Independent samples t-tests,the Chi-square (χ²)test for categorized

### Correlation analysis

Table 3 presents the results of the Pearson correlation analysis. Knowledge demonstrated a significant positive correlation with nurses’ attitudes (*r* = 0.771, *p* < 0.001) and readiness toward telehealth (*r* = 0.372, *p* < 0.001). Moreover, nurses’ attitudes showed a significant positive correlation with readiness toward telehealth (*r* = 0.454, *p* < 0.001).

**Table 3:** Correlation between knowledge, nurses' attitude and readiness toward telehealth among studied nurses (N=250)

**Scales**		**Total score knowledge**	**Total of readiness toward telehealth**
Total score knowledge	r-value		0.372
	P-value		< 0.001*
Total of nurses’ attitude score	r-value	0.771	0.454
	P-value	< 0.001*	< 0.001*

Pearson correlation

*Correlation is significant at the 0.05 level (2-tailed)

### Regression analysis

Tables 4 and 5 present the results of multiple linear regression analyses to identify predictors of nurses’ knowledge, and attitudes toward telehealth, respectively. Higher knowledge about telehealth was a significant positive predictor with attitudes toward telehealth (B = 0.092, P = < 0.001), married nurses (B = 0.387, *P* = 0.027) and higher educational qualification (B = 0.669, *P* < 0.001).

**Table 4:** Multiple linear regression analysis of total knowledge score of nurses and other parameters (N=250)

Predictor Variables	B	Std. Error	Beta	t	*P* value	95.0% Confidence Interval for B		VIF
						Lower Bound	Upper Bound	
Age	−0.004	0.026	−0.018	−0.170	0.865	−0.056	0.047	7.607
Sex	0.030	0.152	0.008	0.196	0.845	−0.270	0.330	1.020
Marital Status	0.387	0.174	0.090	2.228	0.027*	0.045	0.730	1.073
Educational Qualifications	0.669	0.184	0.178	3.639	< 0.001*	0.307	1.032	1.591
Years’ Experience	0.004	0.024	0.018	0.160	0.873	−0.044	0.051	8.180
Total Attitude scale score	0.092	0.006	0.695	14.635	< 0.001*	0.079	0.104	1.500
Total Readiness scale score	0.004	0.007	0.029	0.656	0.513	−0.009	0.017	1.276

Multicollinearity Assessment: All VIF values < 10, indicating acceptable multicollinearity levels

*VIF * Variance inflation factor

Model Summary: R² = 0.636, Adjusted R² = 0.625, F = 60.393

*P value significant

*p*< 0.001

**Table 5:** Multiple linear regression analysis predicting total nurses' attitude toward telehealth (N=250)

Predictor Variables	B	Std. Error	Beta	t	*P* value	95.0% Confidence Interval for B		VIF
						Lower Bound	Upper Bound	
Age	0.164	0.194	0.089	0.843	0.400	−0.219	0.547	7.586
Sex	−0.330	1.138	−0.011	−0.290	0.772	−2.572	1.912	1.019
Marital Status	−2.979	1.299	−0.091	−2.292	0.023*	−5.538	−0.419	1.071
Educational Qualifications	−0.072	1.413	−0.003	−0.051	0.959	−2.855	2.711	1.678
Years’ Experience	−0.324	0.179	−0.197	−1.810	0.071	−0.677	0.029	8.072
Total Readiness scale score	0.212	0.048	0.184	4.427	< 0.001*	0.118	0.307	1.182
Total Knowledge scale score	5.125	0.350	0.675	14.635	< 0.001*	4.436	5.815	1.457

Multicollinearity Assessment: All VIF values < 10, indicating acceptable multicollinearity levels

*VIF*Variance inflation factor

Model Summary: R² = 0.646, Adjusted R² = 0.636, F = 63.157

*P value significant

*p*< 0.001

Regarding nurse’s attitude had significant positive association with total Readiness scale score shows (β = 0.184, *p* < 0.001) and a significant negative relationship (β = −0.091, *p* = 0.023) with married nurses.

The subgroup analysis revealed significant age-related differences across all three domains of knowledge, attitude toward telemedicine, and total readiness (all *p* ≤ 0.004). Younger nurses under 30 years demonstrated the highest readiness scores (66.92 ± 10.23) despite having moderate knowledge levels, while middle-aged nurses between 30 and 40 years exhibited the highest knowledge scores (5.38 ± 1.66). In contrast, older nurses over 40 years consistently scored lowest across all domains.(see supplementary data Table 6).

Similarly, significant differences were observed across hospital settings (all *p* < 0.05) regarding nurses’ knowledge, attitude toward telemedicine, and total readiness. Orman hospital consistently demonstrated the highest performance across all domains with knowledge scores of 5.45 ± 1.84, attitude scores of 64.43 ± 12.45, and readiness scores of 66.23 ± 10.71. Conversely, the Neurology and Psychiatry hospital showed the lowest performance, particularly in knowledge scores (4.65 ± 1.89)(see supplementary data Table 7).

## Discussion

Telehealth has emerged as a fundamental pillar in contemporary nursing practice, transcending geographical constraints and substantially enhancing both the quality and accessibility of healthcare services. Beyond its immediate clinical benefits, telehealth emphasizes sustainable patient well-being and strategically empowers nursing professionals through comprehensive training opportunities, structured follow-up care protocols, and enhanced family support systems. The technological infrastructure encompasses diverse information and communication technology (ICT) systems, including traditional telecommunication tools such as telephones and faxes, sophisticated internet-based platforms, specialized software applications, contemporary social media platforms including Facebook and WhatsApp, advanced voice and video conferencing technologies, and integrated computer systems [31].

The present investigation provides comprehensive insights into the knowledge, attitudes, and readiness of nursing staff toward telehealth implementation within an Egyptian university hospital context. Our findings illuminate significant associations between these interconnected factors, underscoring the pivotal role of knowledge in shaping nurses’ attitudes and readiness for successful telehealth implementation. Statistical analysis revealed significant differences in telehealth knowledge based on age, qualifications, work experience, and hospital affiliation. Notably, nurses over 40 years old were underrepresented in the group with good telehealth knowledge. Conversely, nurses with a degree in nursing and those working in neurology and psychiatric hospitals were more frequently found in the low-skilled group. Interestingly, nurses with over 20 years of experience were also more likely to be in the poor knowledge group.

These findings align with Albarrak et al. (2021), who documented that medical professionals across multiple departments frequently exhibit substantial limitations in telehealth technology knowledge and demonstrate insufficient awareness, attitudes, and technical competencies [2]. However, contrasting findings were reported by Yu-tong et al. (2022), who identified significant variations in telehealth readiness scores contingent upon workplace characteristics and marital status. Their research demonstrated that head nurses achieved superior performance compared to staff nurses, while married nurses significantly outperformed their unmarried colleagues, attributed to the enhanced clinical experience and elevated professional positions typically associated with head nursing roles [5].

Our investigation documented significant inter-group differences regarding nurses’ attitudes and readiness toward telehealth implementation. The group with good knowledge demonstrated more positive attitudes compared to the poor knowledge group. This observation reinforces the fundamental principle that enhanced knowledge directly facilitates improved attitudinal perspectives. This correlation aligns closely with Biruk and Abetu, who reported that healthcare professionals possessing elevated telehealth knowledge levels consistently exhibit positive attitudes toward its implementation [28]. Similarly, Gund et al. found that healthcare professionals with high information levels generally maintain positive attitudes towards current and future electronic communication tools [32]. Dangyang (2017) further corroborated these findings, reporting that all participants in their study demonstrated good knowledge, willingness, and positive attitudes towards telenursing. The participants believed that telenursing would enhance their clinical nursing outcomes and expressed a desire for its implementation [33].

Nevertheless, some investigations present contradictory results. Sheikhtaheri et al. (2016) reported that while the majority of study participants maintained positive attitudes toward telehealth, their knowledge levels were comparatively deficient [34]. Similarly, Keshvari et al. (2015) found that despite weak knowledge levels among most health workers, their attitudes towards telehealth adoption remained positive [35]. Additional research has identified that healthcare professionals’ intentions to utilize telehealth applications are fundamentally influenced byfactors including perceived usefulness, perceived ease of use, and overall attitude. These factors significantly and positively affect behavioral intention to use telehealth, with attitude often mediating the relationship between perceived ease of use and intention [36]. 

The current study revealed that the group with poor telehealth knowledge more frequently exhibited low readiness for telehealth implementation. Total scores and telehealth readiness scales were, on average, higher in the good knowledge group. This finding aligns with Gibson et al. (2020), who reported that clinical nurses lacking adequate telehealth training and knowledge negatively impacted their preparedness for future telehealth careers [37].

Arends et al. (2021) convincingly demonstrated the effectiveness of targeted educational interventions, reporting significant improvements in nurses’ readiness, confidence, and operational ability to utilize telehealth following completion of an evidence-based telehealth curriculum that incorporated practical demonstrations, hands-on utilization, and realistic simulations [38].

Our study documented a statistically significant positive correlation between knowledge levels and nurses’ attitudes and readiness toward telehealth. This finding contrasts with Bashir et al. (2023), who reported that despite positive attitudes and readiness for telehealth, the majority of healthcare professionals still had limited knowledge, emphasizing the critical need for specialized training programs to ensure appropriate implementation and sustainable continuation of telehealth services [29]. Elhadi et al. (2021) presented conflicting results, noting that that most study participants achieved elevated attitude and readiness scores, regardless of variable knowledge levels, stressing the fundamental importance of attitudes and willingness in determining how healthcare professionals perceive telehealth as essential for successful acceptance and comprehensive understanding of telehealth methodologies [39].

These discrepancies may be attributed to significant contextual variations. In well-resourced or digitally advanced healthcare settings, telehealth exposure may occur through robust institutional support or routine system utilization, thereby reducing dependence on individual knowledge acquisition. In contrast, within our Egyptian healthcare context—characterized by limited formal telehealth training and constrained digital infrastructure—knowledge becomes the primary catalyst for both professional acceptance and operational readiness. Furthermore, cultural factors including established professional hierarchies, resistance to technological innovation among senior staff members, and significant gaps in digital literacy may substantially contribute to reduced telehealth engagement among experienced nurses.

Multiple linear regression analyses successfully identified key predictive factors influencing nurses’ telehealth knowledge and attitudes. Enhanced telehealth knowledge emerged as a significant positive predictor of favorable attitudes, alongside marital status and higher educational qualifications as contributing factors. Conversely, nurses’ attitudes demonstrated a statistically significant positive correlation with total readiness scale scores, while exhibiting a significant negative association with married status.

Comprehensive subgroup analysis revealed statistically significant age-related differences across all three evaluated domains: knowledge, attitude toward telemedicine, and overall readiness. Younger nurses (under 30 years) demonstrated the highest readiness scores despite maintaining moderate knowledge levels, while middle-aged nurses (30–40 years) exhibited the highest knowledge scores. In contrast, older nurses (over 40 years) consistently achieved the lowest performance across all evaluated domains. Significant variations were also observed across hospital settings regarding nurses’ knowledge, attitudes toward telemedicine, and total readiness scores. Orman Hospital consistently demonstrated superior performance across all domains with the highest knowledge, attitude, and readiness scores. Conversely, the Neurology and Psychiatry Hospital exhibited the poorest performance, particularly evident in knowledge assessment scores.

The synthesized findings strongly support the conceptualization that nurses’ knowledge, attitude, and readiness constitute interconnected constructs influencing telehealth adoption, thereby reinforcing established theoretical models including the Technology Acceptance Model (TAM) and Theory of Planned Behavior (TPB) [22, 25, 40]. The evidence demonstrating that readiness can motivate self-directed knowledge acquisition aligns with theories emphasizing proactive learning behaviors in technology adoption contexts. The relationship between knowledge and readiness exhibits inherent complexity; higher readiness appears to foster self-directed learning, which subsequently enhances knowledge acquisition. This suggests a reciprocal relationship rather than unidirectional causation, challenging simplistic linear models and supporting more dynamic frameworks of competency development in telehealth contexts [22, 41].

The identification of core competencies required for telehealth nursing—including digital proficiency, effective communication, and ethical considerations—extends existing theoretical frameworks by integrating telehealth-specific professional activities (NT-EPAs), thereby advancing competency-based education theories in nursing practice [24, 42, 43]. The critical role of attitudes, particularly optimism and perceived usefulness, as psychological antecedents to telehealth acceptance corroborates and extends the Technology Readiness Model. These findings highlight the fundamental importance of affective and cognitive factors in telehealth uptake among nursing professionals [25, 44].

The findings emphasize the paramount importance of contextual and organizational readiness factors, including facilitating conditions and social influence, which interact dynamically with individual readiness and knowledge levels. This evidence supports multi-level theoretical models of technology adoption in healthcare settings [45, 46]. The evidence that demographic factors (age, experience) moderate technology acceptance and readiness indicates that theoretical models must incorporate individual differences and socio-technical dynamics to comprehensively explain telehealth adoption patterns among nursing professionals [25, 41, 44].

## Limitations

This study has several limitations that should be considered when interpreting the results. The use of convenience sampling may introduce selection bias and limit the generalizability of the findings to the broader nursing population. Additionally, the cross-sectional nature of the study limits the ability to establish causality between nurses’ knowledge, attitudes, and readiness toward telehealth. Although significant associations were found, it remains unclear whether increased knowledge leads to improved attitudes and readiness or vice versa. Furthermore, the use of self-administered questionnaires may have introduced social desirability bias, as participants might have provided responses they perceived as more favourable, particularly regarding their attitudes and willingness to adopt telehealth practices. In addition, the study was conducted in a specific institutional context, which may not reflect broader national or international settings. Future research should consider longitudinal designs and more diverse samples to validate and expand upon these findings. Lastly, Content validity of tools was assessed through expert panel review using a qualitative consensus approach. Although formal Content Validity Index (CVI) or Content Validity Ratio (CVR) calculations were not conducted, all five experts independently confirmed the relevance, clarity, and cultural appropriateness of each instrument item. Future research is encouraged to employ quantitative validity measures to enhance the methodological rigor and replicability of instrument development.

Practical implications: The study highlights the urgent need for healthcare systems to embed structured, competency-based telehealth education into nursing practice as a foundation for fostering positive attitudes and implementation readiness. Tailored training programs should address digital literacy gaps—especially among older or traditionally trained nurses—by incorporating experiential learning, clinical simulations, and communication protocols specific to virtual care. To ensure sustainable adoption, organizations must invest not only in user-friendly technologies but also in supportive policies, leadership engagement, and mentorship models that ease resistance to change. Integrating telehealth competencies into all levels of nursing education, alongside gender-sensitive approaches and ongoing skill assessments, is essential. These strategies should be backed by policy frameworks that promote continuous evaluation and improvement to achieve long-term, system-wide integration of telehealth in nursing care.

The practical relevance of these findings gains additional significance through evidence demonstrating that successful telehealth implementation in chronic disease management contexts has been consistently linked to healthcare providers’ comprehensive knowledge base, favorable attitudes, and operational readiness [47, 48]. This broader contextual perspective reinforces the current research findings and emphasizes the fundamental importance of targeted capacity-building initiatives and comprehensive training programs among nursing personnel to facilitate effective telehealth integration across diverse healthcare settings.

## Conclusion

The findings of this study underscore the strong influence of demographic and professional factors—such as age, qualifications, experience, and hospital setting—on nurses’ telehealth knowledge. Notably, older and more experienced nurses, as well as those in specific clinical areas like neurology and psychiatry, were more likely to have lower knowledge levels. This knowledge gap was significantly associated with less favourable attitudes and lower readiness for telehealth implementation. In contrast, nurses with higher knowledge demonstrated more positive perceptions and greater preparedness to engage in telehealth practices. These results highlight the central role of knowledge in shaping both the mindset and practical readiness of nursing staff, reinforcing the need for targeted educational strategies to bridge gaps and ensure effective integration of telehealth into nursing care. 

## Supplementary Information

Supplementary material 1. Subgroup analysis by age group with nurse’s knowledge, attitude toward telehealth and readiness (n=250).

Supplementary material 2. Subgroup analysis by type of hospital with nurse’s knowledge, attitude toward telehealth and readiness (n=250).

## Data Availability

All data generated or analyzed during this study are available from corresponded on request.

## Abbreviations

TRA: Telehealth readiness assessment tool
