# Supplementary material for: Knowledge, attitude and readiness toward telehealth among nursing staff: a cross-sectional study
Source: BMC Med Educ. 2025 Oct 21;25:1448. doi: 10.1186/s12909-025-07921-1 (PMC12539162; doi:10.1186/s12909-025-07921-1)
Supplement: Supplementary file 1 — Supplementary material 1. Subgroup analysis by age group with nurse’s knowledge, attitude toward telehealth and readiness (n=250). [file 12909_2025_7921_MOESM1_ESM.docx]

**This questionnaire is to asses Health Professionals knowledge and attitude towards telemedicine.**

**You are selected randomly to participate in this study and your participation is purely based on your willingness. You have the right to choose not to take part in this study and you have the right to stop at any time. All information will be recorded anonymously so please don't write your name or any identification.**

**Thank you for your time!**

**Health Professionals knowledge and attitude assessment on telemedicine questionnaire**

**First part: Sociodemographic information of the health professionals**

1. **Age**
2. **Sex: 1-male 2-female**
3. **Education status :**

**1-diploma degree 2- masters 3-others**

1. **Type of profession :**

**1-Physician 2-Nurse 3- Health officer 4-Medical laboratory 5-Pharmacist 6-midwifery 7-others**

1. **Year of experience:**

**1- <5 years**

**2- 5-10 years**

**3- >10 years**

1. **Salary:**

**1- <1500**

**2- 1500-3500**

**3- 3500-5500**

**Second part:**

**Table 2. Health professionals Knowledge toward telemedicine technology**

| **NB** | **QUESTION** | **Answers** | |
| --- | --- | --- | --- |
| **1** | **Have you ever heard about telemedicine** | **yes** | **no** |
| **2** | **If yes, what was your source of information?** | **Training**  **Public media**  **Internet**  **Colleagues**  **others** | |
| **3** | **Have you ever seen telemedicine system?** | **yes** | **no** |
| **4** | **I know telemedicine technology** | **yes** | **no** |
| **5** | **I know telemedicine tools like tele surgery, teleconsultation, teleconferencing and so on** | **yes** | **no** |
| **6** | **I know the effect of telemedicine on healthcare quality** | **yes** | **no** |
| **7** | **I know the effect of telemedicine on reducing medical staffs needed** | **yes** | **no** |
| **8** | **I know about telemedicine infrastructure** | **yes** | **no** |
| **9** | **I know the benefits of telemedicine on reducing the unnecessary transportation cost** | **yes** | **no** |
| **10** | **I know the benefits of telemedicine in saving clinicians time** | **yes** | **no** |

**Table 3. Health professionals’ attitude toward telemedicine technology**

| **No** | **I believe that telemedicine may ….** | **strongly disagree** | **disagree** | **Neutral** | **agree** | **strongly agree** |
| --- | --- | --- | --- | --- | --- | --- |
|  | **Relative advantage** |  |  |  |  |  |
| **12** | **Reduce medical errors** |  |  |  |  |  |
| **13** | **Facilitate diagnosis and treatment** |  |  |  |  |  |
| **14** | **Increase communication among health care providers** |  |  |  |  |  |
| **15** | **telemedicine can reduce the number of visits to healthcare centers** |  |  |  |  |  |
| **16** | **Enables me accomplish my task more quickly** |  |  |  |  |  |
| **17** | **Improve clinical decisions** |  |  |  |  |  |
| **18** | **Provide more comprehensive healthcare services** |  |  |  |  |  |
|  | **compatibility** |  |  |  |  |  |
| **19** | **In my opinion, telemedicine is compatible with all aspects of my work** |  |  |  |  |  |
| **20** | **telemedicine is completely compatible with my current situation** |  |  |  |  |  |
| **21** | **I think telemedicine fits well with the way I like to work** |  |  |  |  |  |
| **22** | **Using telemedicine fits well into my work style** |  |  |  |  |  |
|  | **complexity** |  |  |  |  |  |
| **23** | **I believe using telemedicine requires a lots of mental effort *** |  |  |  |  |  |
| **24** | **Learning to operate telemedicine is hard for me*** |  |  |  |  |  |
| **25** | **I think telemedicine Increase staff work load *** |  |  |  |  |  |
| **26** | **I think telemedicine Create new responsibilities for staff*** |  |  |  |  |  |
| **27** | **In my opinion, telemedicine Threaten information confidentiality and patient privacy*** |  |  |  |  |  |
|  | **Trial ability** |  |  |  |  |  |
| **28** | **I believe to try telemedicine applications is a great opportunity** |  |  |  |  |  |
| **29** | **I do not have to take very much effort to try out telemedicine** |  |  |  |  |  |
| **30** | **I believe, using telemedicine on a trial basis is enough to see what it could do** |  |  |  |  |  |
| **31** | **I would like to try out telemedicine applications before using it** |  |  |  |  |  |
|  | **observability** |  |  |  |  |  |
| **32** | **I have seen what other hospital staffs do with telemedicine** |  |  |  |  |  |
| **33** | **telemedicine is very visible in the hospital where I work** |  |  |  |  |  |
| **34** | **In the hospital, I see telemedicine being used for many tasks** |  |  |  |  |  |

**reverse scored (1=strongly agree ….5=strongly disagree)*
