# Supplementary material for: Knowledge, attitude and readiness toward telehealth among nursing staff: a cross-sectional study
Source: BMC Med Educ. 2025 Oct 21;25:1448. doi: 10.1186/s12909-025-07921-1 (PMC12539162; doi:10.1186/s12909-025-07921-1)
Supplement: Supplementary file 2 — Supplementary material 2. Subgroup analysis by type of hospital with nurse’s knowledge, attitude toward telehealth and readiness (n=250). [file 12909_2025_7921_MOESM2_ESM.docx]

**Supplementary data**

**Table (6) Subgroup Analysis by Age Group with nurse’s Knowledge, attitude toward telehealth and readiness (N=250)**

**(N=250)**

| **Age Group** | **Total of Knowledge Score scale** | **Total of Attitude Score scale** | **Total of Readiness Score scale** |
| --- | --- | --- | --- |
| **< 30 years**  (N=64) | 5.04±1.62 | 63.09±13.05 | 66.92±10.23 |
| **30-40 years**  (N=126) | 5.38±1.66 | 62.84±13.52 | 63.17±12.46 |
| **> 40 years**  (N=60) | 4.28±1.96 | 55.60±12.75 | 59.91±10.67 |
| **P-value** | **<0.0001** | **0.001** | **0.004** |

***All subgroup analyses conducted using ANOVA with post-hoc tests***

**Table (7): Subgroup Analysis by type of Hospital with nurse’s Knowledge, attitude toward telehealth and readiness (N=250)**

| **Type of Hospital** | **Total of Knowledge Score scale** | **Total of Attitude Score scale** | **Total of Readiness Score scale** |
| --- | --- | --- | --- |
| **Neurology and Psychiatry hospital (N=86)** | 4.65±1.89 | 58.77±14.71 | 61.27±12.32 |
| **Orman hospital (N=82)** | 5.45±1.84 | 64.43±12.45 | 66.23±10.71 |
| **Rajehy hospital(N=82)** | 5.03±1.78 | 60.41±12.80 | 62.64±11.62 |
| **P-value** | **0.014*** | **0.020*** | **0.018** |

***All subgroup analyses conducted using ANOVA with post-hoc tests***
